# Osteopathic Manipulative Treatment for Asthma: A Systematic Review of Objective Pulmonary Function Outcomes

**DOI:** 10.7759/cureus.105000

**Published:** 2026-03-10

**Authors:** Alexander Ponce, Oliver Perrine, Kevin A Heintzelman

**Affiliations:** 1 Internal Medicine, William Carey University College of Osteopathic Medicine, Hattiesburg, USA; 2 Internal Medicine, Baptist Memorial Health Care, Jackson, USA

**Keywords:** asthma, omm, omt, osteopathic manipulative treatment, osteopathic medicine, reactive airway disease

## Abstract

Asthma is a common obstructive airway disease associated with significant functional impairment and morbidity. Osteopathic manipulative treatment (OMT) has been proposed as an adjunctive therapy to address musculoskeletal and respiratory mechanical dysfunctions associated with asthma; however, supporting evidence remains limited. This systematic review evaluates whether OMT, compared with sham treatment or usual care, improves pulmonary function outcomes in patients with asthma, as measured by peak expiratory flow (PEF) and spirometric indices including forced expiratory volume in 1 second (FEV₁) and forced vital capacity (FVC). A systematic literature search of MEDLINE/PubMed, Google Scholar, Cochrane Library, and Semantic Scholar databases was conducted to identify clinical studies evaluating OMT in patients with asthma. Five clinical studies met the inclusion criteria and were included in the qualitative synthesis. Sample sizes were small across studies, and there was substantial heterogeneity in study design, intervention protocols, and outcome measures. While some studies reported improvements in pulmonary function following OMT, the overall risk of bias was moderate, and the certainty of evidence was low to moderate. In conclusion, available evidence suggests that OMT may be associated with improvements in pulmonary function in patients with asthma; however, these findings should be interpreted cautiously due to limited sample sizes, heterogeneity, and methodological limitations. Larger, well-designed randomized controlled trials are needed to more definitively determine the role of OMT in the management of asthma.

## Introduction and background

Asthma is a prevalent chronic respiratory disease characterized by variable airflow obstruction, airway hyperresponsiveness, and underlying airway inflammation [1]. In the United States, asthma affects approximately 24 million individuals and remains one of the most common chronic illnesses of childhood. The disease disproportionately impacts socioeconomically disadvantaged populations and contributes to significant morbidity and mortality [1]. Objective pulmonary function testing, including spirometry and peak expiratory flow (PEF), plays a central role in the diagnosis, monitoring, and assessment of disease severity in both pediatric and adult patients [2].

Current asthma management follows a stepwise, pharmacologic approach aimed at relieving acute bronchoconstriction and controlling chronic airway inflammation [3]. Short-acting beta-agonists are used for symptom relief during exacerbations, while inhaled corticosteroids form the foundation of long-term disease control [3]. Additional therapies, including long-acting bronchodilators and other controller medications, may be incorporated based on symptom burden and disease severity [1]. Although these strategies are effective for many patients, asthma often requires lifelong therapy, and prolonged medication use may be associated with adverse effects, particularly in pediatric populations [4].

Given the chronic nature of asthma and the burden associated with long-term pharmacologic management, interest has grown in adjunctive, non-pharmacologic interventions. However, prior evaluations of such approaches have yielded inconsistent and inconclusive results, in part due to heterogeneity in study design, patient populations, and outcome measures. Pulmonary rehabilitation represents one non-pharmacologic intervention that has demonstrated benefit in asthma management; through structured exercise training, it aims to improve physical conditioning and has been associated with gains in inspiratory muscle strength and other objective pulmonary measures [5]. In contrast, other non-pharmacologic approaches, such as chiropractic care, may provide subjective symptom improvement without corresponding significant changes in objective lung function [6]. This variability has made it difficult to determine whether observed benefits reflect true physiologic improvement or context-dependent effects.

Osteopathic manipulative treatment (OMT) has been proposed as a potential adjunctive therapy in asthma management. OMT is theorized to influence thoracic mechanics, chest wall mobility, and respiratory effort -- factors that may plausibly affect pulmonary function measures, particularly effort-dependent outcomes such as PEF [7]. Clinical studies evaluating OMT in asthma have reported mixed findings, and the extent to which OMT influences objective pulmonary function remains unclear. A prior review published in 2016 concluded that OMT represents a safe, non-pharmacologic resource for asthma management [8]. Since that time, additional studies evaluating OMT in patients with asthma have been published, warranting reassessment of this intervention in light of newer evidence. Accordingly, this systematic review aims to evaluate whether OMT, compared with sham treatment or usual care, improves pulmonary function in patients with asthma as measured by PEF and spirometric indices (forced expiratory volume in 1 second (FEV₁), forced vital capacity (FVC), and FEV₁/FVC).

## Review

Materials and methods

Search Strategy

A comprehensive literature search was conducted to identify clinical studies evaluating the use of OMT in patients with asthma. The databases MEDLINE/PubMed, Google Scholar, the Cochrane Library, and Semantic Scholar were searched from database inception through January 16th, 2026. Search strategies were adapted for each database to account for differences in indexing systems, controlled vocabulary, and search functionality while maintaining consistent conceptual domains related to asthma and OMT.

Search terms included combinations of keywords and controlled vocabulary related to osteopathy and asthma, such as “osteopathic manipulative treatment,” “osteopathic manipulation,” “osteopathic medicine,” and “asthma.” In MEDLINE/PubMed, both free-text terms and Medical Subject Headings (MeSH) were used to optimize sensitivity. Google Scholar searches prioritized articles with high topical relevance by limiting queries to titles when appropriate. The Cochrane Library and Semantic Scholar were searched using comparable keyword combinations to ensure comprehensive retrieval of relevant studies.

Reference lists of included articles were also reviewed to identify additional studies that may not have been captured in the initial database searches. The complete search strategy was designed to maximize the identification of relevant clinical trials evaluating the effects of OMT on pulmonary function outcomes in patients with asthma.

Eligibility Criteria

Studies were eligible for inclusion if they involved patients with asthma, evaluated OMT as an intervention, and reported patient-level clinical or physiological outcomes. All clinical studies evaluating OMT in patients with asthma were eligible for inclusion, regardless of study design, clinical context, or outcome measure. Narrative reviews, systematic reviews, and studies evaluating non-osteopathic manual therapies were excluded.

Study Selection and Data Extraction

**Figure 1:**
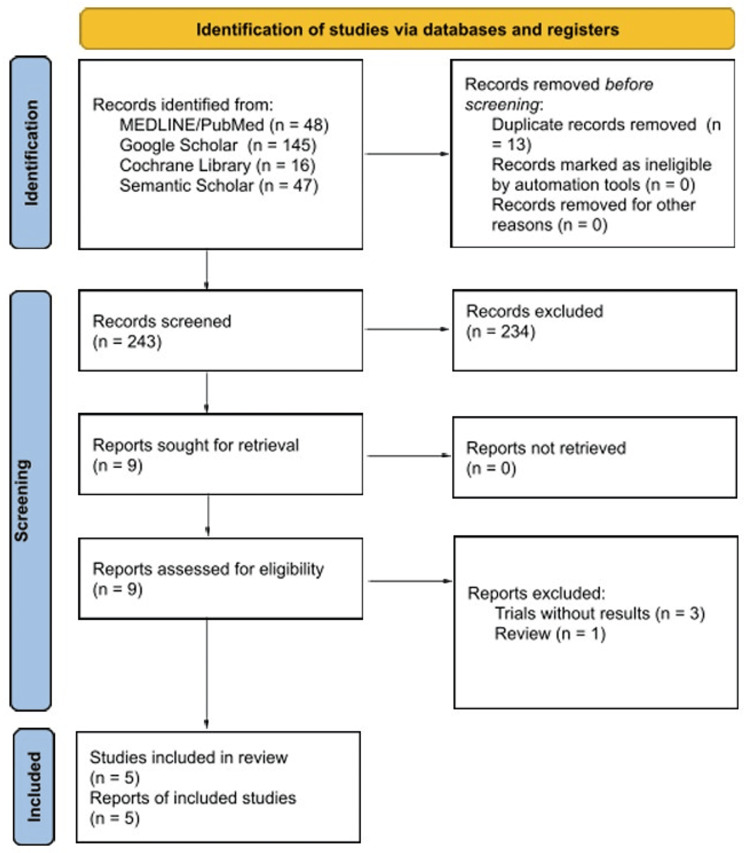
PRISMA 2020 flow diagram illustrating the study selection process PRISMA, Preferred Reporting Items for Systematic Reviews and Meta-Analyses.

Literature searches and study selection were performed by two authors (A.P. and O.P.). Any discrepancies between reviewers were resolved by consensus; however, no disagreements occurred during study selection or data extraction. Risk-of-bias assessments were applied at the study level using predefined criteria. Titles and abstracts were screened for relevance, followed by full-text review of potentially eligible studies. Reasons for exclusion at the full-text stage are detailed in the Preferred Reporting Items for Systematic Reviews and Meta-Analyses (PRISMA) 2020 flow diagram (Figure 1) [9]. For included studies, data were extracted regarding study design, sample size, patient population, outcomes reported, key findings, primary limitations, and risk of bias. Due to heterogeneity in study design and outcomes, results were synthesized narratively rather than pooled for meta-analysis. Study selection is summarized in Figure 1. This systematic review was conducted and reported in accordance with the PRISMA 2020 guidelines [9].

Results

A total of 256 records were identified through the initial literature search. After screening, nine articles were sought for retrieval and assessed for eligibility. These nine articles underwent full-text review, and five studies were ultimately included in the review. Pulmonary function outcomes assessed across the included studies included spirometric measures -- FEV₁, FVC, the FEV₁/FVC ratio, and forced expiratory flow at 25-75% of vital capacity (FEF 25-75%) -- as well as PEF.

Three trials were excluded due to the absence of reported results, and one paper was excluded as it represented a prior review of the topic. While many articles mentioned asthma or OMT, few evaluated OMT as an intervention for asthma using objective pulmonary function testing. Summaries of the five included studies, including study design, sample size, patient population, OMT techniques used, outcomes reported, key findings, primary limitations, and risk of bias, are presented below.

Adult asthma studies evaluating OMT and pulmonary function outcomes are summarized in Table 1. Pediatric asthma studies evaluating OMT are summarized in Table 2. Risk of bias assessments for the randomized controlled trials, using the Cochrane Risk of Bias 2 tool, are presented in Table 3 [10]. Most randomized controlled trials were rated as having “some concerns” for overall risk of bias, primarily related to limitations in randomization procedures, deviations from intended interventions, and outcome measurement. In particular, incomplete reporting of allocation concealment and limited blinding contributed to these ratings despite low risk of missing outcome data. Methodological quality assessments for the uncontrolled pre-post studies, based on National Institutes of Health criteria, are shown in Table 4. Studies rated as “fair” quality generally met criteria for clearly defined objectives and outcome reporting but were limited by small sample sizes, lack of blinding, and incomplete reporting of representativeness and follow-up, which constrains interpretation of observed effects.

**Table 1:** Study design, population, sample size, outcomes measured, and key findings of the adult asthma studies OMT, osteopathic manipulative treatment; PEF, peak expiratory flow; FEV₁, forced expiratory volume in 1 second; FVC, forced vital capacity; FEF 25-75%, forced expiratory flow at 25-75% of vital capacity.

Author	Study Design	Population	Sample Size	OMT Techniques Used	Relevant Outcomes Measured	Key Findings
Bockenhauer et al. [11]	Randomized control trial	Adult patients with asthma	n = 10	Balanced ligamentous tension in the occipitoatloid and the cervicothoracic junctions, A.T. Still technique for “upward displacement” of the first rib, direct action release of “lower rib exhalation restriction,” and diaphragmatic release	PEf	Mean PEF showed a decrease after sham and OMT, but not to a statistically significant degree
Kasten et al. [12]	Single-arm clinical trial	Adult patients with asthma	n = 25	Supine occipitoatlantal (OA) joint balanced ligamentous tension (BLT), supine cervical spine Still technique, supine thoracic inlet myofascial release (MFR), stimulatory supine rib raising soft tissue, supine rib BLT, supine abdominal diaphragm MFR, seated thoracic spine Still technique, and seated posterior rib Still technique	Spirometry (FEV_1_, FVC, FEV_1_/FVC), PEF	Only the FEV1/FVC ratio of spirometry values was statistically significant. In addition, there was a statistically significant improvement in PEF
Ragland [13]	Randomized control trial	Adult patients with asthma	n = 24	Thoracic spine soft tissue, rib raising, doming of the diaphragm myofascial release, cervical spine soft tissue, suboccipital decompression, thoracic inlet myofascial release, and thoracic lymphatic pump with activation	Spirometry (FEV_1_, FVC, FEV_1_/FVC)	No statistically significant changes in spirometry. A small change in FEV_1_/FVC ratio between pre- and post-OMT change of 1.15% was noted, this change lacks statistical significance and was not accompanied by any substantive changes in FEV_1_ or FVC

**Table 2:** Study design, population, sample size, outcomes measured, and key findings of the pediatric asthma studies OMT, osteopathic manipulative treatment; PEF, peak expiratory flow; FEV₁, forced expiratory volume in 1 second; FVC, forced vital capacity; FEF 25-75%, forced expiratory flow at 25-75% of vital capacity.

Author	Study Design	Population	Sample Size	OMT Techniques Used	Outcomes Measured	Key Findings
Jones et al. [14]	Randomized control trial	Children with asthma aged 7-18 years	n = 58	Rib raising and suboccipital release	Spirometry (FEV_1_, FVC, FEV_1_/FVC, and FEF 25-75%)	No statistically significant changes in FEV_1_, FVC, FEV_1_/FVC ratio, and FEF 25-75%. Moderate changes with improved FEV_1_, FVC, FEV_1_/FVC ratio, and FEF 25-75% were observed, but not to a degree of statistical significance
Guiney et al. [15]	Randomized control trial	Children with asthma aged 5-17 years	n = 140	Rib raising, muscle energy for ribs, and myofascial release	PEF	Statistically significant improvement in PEF when comparing OMT group to sham treatment control group (p < 0.05). PEF mean difference following treatment when compared to control was 12.7 L per minute

**Table 3:** Cochrane RoB2 tool for randomized control trials included in the study RoB, risk of bias.

Study	RoB2 Applicable?	Randomization?	Deviations From Intended Interventions	Missing Outcome Data	Outcome Measurement	Selection of Reported Result	Overall RoB
Jones et al. [14]	Yes	Low	Some concerns	Low	Low	Some concerns	Some concerns
Guiney et al. [15]	Yes	Some concerns	Some concerns	Low	Some concerns	Some concerns	Some concerns
Bockenhauer et al. [11]	Yes	Some concerns	Some concerns	Low	Some concerns	Some concerns	Some concerns

**Table 4:** NIH pre-post table for the uncontrolled studies included in the review NIH, National Institutes of Health.

NIH Item	Kasten et al. [12]	Ragland [13]
1 Objective stated	Yes	Yes
2 Eligibility described	Yes	Yes
3 Representativeness	Cannot determine	Cannot determine
4 All eligible enrolled	Not reported	Not reported
5 Sample size justification/power	Not reported	Not reported
6 Intervention clearly delivered	Yes	Yes
7 Outcomes prespecified/valid	Yes	Yes
8 Blinded assessors	Not reported	Not reported
9 Follow-up rate ≥80%	Cannot determine	Cannot determine
10 Appropriate statistics	Yes	Yes
11 Multiple pre/post measures	Yes	Yes
12 Pre/post results reported	Yes	Yes
Overall quality	Fair	Fair

Discussion

This systematic review evaluated the efficacy of OMT as a supplemental intervention in adult and pediatric patients with asthma, with a focus on objective pulmonary function outcomes. Findings from the five included studies suggest that OMT may offer context-dependent benefits; however, the overall evidence remains heterogeneous due to differences in study methodology. This heterogeneity is reflected in the reported outcomes for spirometry and PEF and limits the certainty of observed effects.

Spirometry

Spirometry was the primary method used to assess pulmonary function across the included studies, although it was not employed in every trial. Kasten et al. reported statistically significant changes in spirometric outcomes, specifically a decrease in the FEV₁/FVC ratio from 0.81 to 0.80 between week 1 and week 7 [12]. While statistically significant, this magnitude of change is small and likely of limited clinical relevance. Other spirometric parameters demonstrated numerical differences that did not reach statistical significance, including FEV₁ (3.60 L vs. 3.57 L) and FVC (4.45 L vs. 4.49 L). Ragland reported no statistically significant changes in FEV₁, FVC, or the FEV₁/FVC ratio following OMT, although a small numerical change in the FEV₁/FVC ratio was observed between pre- and post-treatment measurements [13]. Jones et al. similarly reported no statistically significant changes in FEV₁, FVC, FEV₁/FVC ratio, or forced expiratory flow at 25-75% of vital capacity (FEF₂₅-₇₅%) in pediatric patients with asthma [14]. While numerical differences between OMT and control groups were observed for several spirometric measures, these findings were inconsistent and did not demonstrate a clear or reproducible pattern of improvement.

Collectively, spirometric outcomes following OMT were inconsistent and predominantly did not reach statistical significance across studies. Observed changes were generally small and, where statistically significant, unlikely to represent clinically meaningful improvements in fixed airflow obstruction or inflammation-mediated airflow limitation. These findings suggest that any potential effects of OMT on spirometric measures may be limited or context-dependent. Interpretation is further constrained by small sample sizes and limited blinding across studies, which increase susceptibility to performance and detection bias. Overall, the current evidence does not support a clear or consistent effect of OMT on standard spirometric parameters in either adult or pediatric patients with asthma.

Peak Expiratory Flow

PEF was one of the most commonly assessed pulmonary function measures across the included studies and was used to evaluate changes in airflow obstruction in patients with asthma. Kasten et al. reported statistically significant improvements in mean PEF, increasing from 7.43 L to 7.78 L from baseline to after seven weeks of OMT [12]. Similarly, Guiney et al. demonstrated a statistically significant improvement in PEF, with a greater mean change observed in the OMT group compared with the control group (13 vs. 0.3) [15]. The percent change in PEF was 4.8% in the OMT group compared with 1.4% in the control group. 

In contrast, Bockenhauer et al. reported numerical differences in PEF between the OMT and sham treatment groups (−14 vs. −2.5); however, these differences did not reach statistical significance [11]. When considered collectively, findings related to PEF following OMT were heterogeneous and inconsistent across studies [11,12,15]. Although some trials reported statistically significant changes, these effects were modest in magnitude and not uniformly reproduced, making their clinical relevance uncertain.

Given the effort-dependent nature of PEF, observed changes may reflect variability in patient effort, chest wall mechanics, or respiratory muscle performance rather than durable improvement in fixed airway obstruction. Effort-dependent outcomes are also more susceptible to performance effects, particularly in the context of manual therapy interventions. Combined with small sample sizes, variable treatment protocols, and inconsistent findings across trials, these factors limit confidence in the clinical significance of observed PEF changes. Accordingly, current evidence remains inconclusive regarding the effectiveness of OMT for improving PEF in both adult and pediatric patients with asthma.

Limitations

Several methodological limitations were identified among the included studies that limit the strength and generalizability of the findings. Most studies were underpowered due to small sample sizes, reducing statistical sensitivity. Additionally, substantial heterogeneity was present in study design, including differences in patient populations, baseline asthma severity, background pharmacologic therapy, number of treatment sessions, and duration of follow-up. In some included studies, outcome assessment was limited to immediate pre-post intervention measurements without a clearly defined follow-up period; for example, Guiney et al. assessed PEF only immediately before and after a single treatment session, precluding evaluation of durability or sustained clinical effect [15]. OMT protocols varied considerably across studies and were often poorly described. Several studies employed individualized, non-standardized treatment approaches without sufficient detail to allow reproducibility. Furthermore, outcome measures were inconsistent, with some studies relying exclusively on PEF while others incorporated spirometric indices, limiting comparability across trials.

Risk of bias was an important consideration across included studies. Manual therapy interventions are inherently difficult to blind, increasing susceptibility to performance and detection bias, particularly for effort-dependent outcomes such as PEF. Small sample sizes and limited allocation concealment further constrain interpretation of reported effects. These factors collectively reduce confidence in the observed findings and limit conclusions regarding clinical significance. This systematic review also has inherent limitations. The inclusion of both adult and pediatric populations may limit generalizability. Variability in OMT techniques, treatment frequency, and intervention duration contributed to clinical and methodological heterogeneity, precluding quantitative synthesis. Additional limitations include the inability to assess publication bias given the small number of included studies and the narrative synthesis approach. Formal grading of certainty of evidence (e.g., GRADE) was not performed due to heterogeneity and limited study numbers, which further constrains confidence in effect estimates.

## Conclusions

In this systematic review, evidence evaluating the effect of OMT on objective pulmonary function outcomes in patients with asthma was limited and heterogeneous. Across five clinical studies, improvements in spirometric indices were inconsistent and generally did not demonstrate a clinically meaningful improvement in either adult or pediatric populations. In contrast, PEF demonstrated more frequent improvement following OMT, although findings varied across studies and were constrained by small sample sizes and methodological differences.

Overall, the current body of evidence does not allow for definitive conclusions regarding the effect of OMT on standard pulmonary function outcomes in asthma. While observed changes in effort-dependent measures, such as PEF, suggest a possible mechanical influence of OMT, the overall certainty of evidence remains low. Larger, well-designed randomized controlled trials with standardized OMT protocols and clearly defined, clinically meaningful endpoints are needed before OMT can be recommended as an adjunctive therapy in asthma management.

## Appendices

Detailed search strategy

Semantic Scholar

The Semantic Scholar database was searched using the following keywords: “osteopathic medicine,” “osteopathic manipulative treatment,” and “asthma.”

Google Scholar

Google Scholar was searched using the following query to identify articles with high topical relevance: intitle:"osteopathic manipulative" asthma

Cochrane Library

The Cochrane Library was searched using the following terms: “osteopathic” AND “asthma.”

MEDLINE/PubMed

MEDLINE/PubMed was searched using a combination of free-text terms and Medical Subject Headings (MeSH) as follows: (osteopath*[tiab] OR "osteopathic manipulation"[tiab] OR "osteopathic manipulative"[tiab] OR "osteopathic manual"[tiab] OR "Manipulation, Osteopathic"[MeSH]) AND (asthma[tiab] OR "Asthma"[MeSH])
